# Dr. Sidney Farber (1903-1973): Founder of Pediatric Pathology and the Father of Modern Chemotherapy

**DOI:** 10.7759/cureus.68286

**Published:** 2024-08-31

**Authors:** Amitabh Kumar Upadhyay, Aaditya Prakash, Abhishek Kumar, Sanghamitra Jena, Neetesh Sinha, Swati Sharma

**Affiliations:** 1 Medical Oncology, Tata Main Hospital, Jamshedpur, IND; 2 Radiation Oncology, Tata Main Hospital, Jamshedpur, IND; 3 Nuclear Medicine, Tata Main Hospital, Jamshedpur, IND; 4 Surgical Oncology, Tata Main Hospital, Jamshedpur, IND; 5 Dentistry, Tata Main Hospital, Jamshedpur, IND

**Keywords:** historical vignette, jimmy fund, aminopterin, modern chemotherapy, pediatric pathology, sidney farber

## Abstract

Dr. Sidney Farber (Farber) was a distinguished pediatric pathologist and widely recognized pioneer of modern chemotherapy. In 1948, his influential study showed that various anti-folates, particularly 4-aminopteroylglutamic acid, also known as aminopterin, induced transient disease control in kids who had acute undifferentiated leukemia. The findings laid the basis for developing and using additional chemotherapies, individually or in combination, for treating pediatric and adult cancers. Farber also introduced actinomycin D to treat Wilms tumor in various stages. Underneath his oversight, the 'Jimmy Fund,' one of the earliest dedicated pediatric oncology centers, and the Children's Cancer Research Foundation, which subsequently evolved into the Dana-Farber Cancer Institute, was established. Farber is known as the "Founder of Pediatric Pathology" and the "Father of Modern Chemotherapy," citing his immense contributions. This article is a tribute to the great scientist Farber for his significant contributions to the scientific field and the countless individuals he has impacted.

## Introduction and background

In the mid to late 1940s, the understanding and treatment options for pediatric oncology, particularly childhood leukemia, were notably limited. Comprehending leukemia etiology and its manifestations was rudimentary, classifying the disease as acute or chronic with a dearth of knowledge about distinct biological subtypes [1]. Diagnostic modalities were restricted to rudimentary techniques such as morphology and histochemistry, with X-rays constituting the primary imaging methodology. The understanding of cell cycle kinetics was minimal, and advanced techniques, like immunophenotyping, molecular genetics, and chromosomal analysis, were unavailable. The roles of signal transduction pathways, growth factor receptors, and receptor tyrosine kinases remained largely enigmatic. Effective primary therapy for acute leukemia was absent, leading to a brief median survival period following diagnosis. Supportive care was primarily limited to red blood cell transfusions and antibiotics [1].

Dr. Sidney Farbers's (Farber) remarkable achievement of attaining the first clinical remission in childhood leukemia through chemotherapy marked a significant milestone in the field [2]. He was widely recognized for his composed and confident demeanor, often described as grandfatherly and gentle, particularly in his interactions with children. His peers deeply respected his understated yet occasionally wry sense of humor, tireless work ethic, and exceptional ability to adapt to diverse circumstances. Farber exuded an air of authority, being acknowledged for his impeccable attire and commanding presence. He ardently advocated for sustained research, adequate funding, and national dedication as fundamental prerequisites for defeating cancer [3-7]. Despite this, his monumental contributions to pediatric pathology and oncology have received relatively lesser recognition than he deserved. This review article stands as a tribute to Farber's extraordinary influence on the lives of millions through his trailblazing research.

## Review

Farber's life and career

Farber, born in Buffalo, New York, on September 30, 1903, to Simon and Matilda Farber, played a significant role in the field of oncology. He entered into matrimony with Norma Farber, a poet, author, and classical soprano in 1928. In 1923, Farber completed his undergraduate studies at the University of Buffalo, New York. He began his medical education in Germany at the Universities of Heidelberg and Freiburg before graduating from Harvard Medical School in 1927. Following this, he underwent further training in pathology at Peter Bent Brigham Hospital in Boston, Massachusetts, under Kenneth Blackfan's guidance [3-7].

After completing his postgraduate training, Farber commenced his tenure at Harvard Medical School in 1929 as an Instructor in Pathology, assuming the esteemed position of the institution's first full-time pathologist at Children's Hospital within the same year. He developed a close mentorship and amicable relationship with pathologist Simeon Burt Wolbach during this period. In 1946, Farber undertook the pivotal position of Chairman of research and laboratory at Children's Hospital Medical Center (CHMC), and a year later, he was appointed as both Pathologist-in-Chief at CHMC and Chairman of the Staff. Revered for his precision and scrupulousness as a scientist, Farber maintained a laboratory distinguished for its immaculate orderliness. He was subsequently appointed Professor of Pathology at Harvard Medical School in 1948 [3-7]. Regrettably, he succumbed to cardiac arrest while on duty on March 30, 1973, leaving behind his wife, four children, and three grandchildren [3-7].

The Jimmy Fund

In 1947, the Variety Club of New England, founded by entertainment community members, recognized Farber as the local scientist whose innovative work inspired financial support and gave rise to the Children's Cancer Research Foundation (CCRF). Initially, the fund's assistance was limited to outpatient only. In May 1948, at the CCRF, the renowned program on radio known as 'Truth or Consequences' featured 'Jimmy,' who was a cancer patient about 12 years of age. Farber and Bill Koster, a member of the Variety Club, scouted the wards of Children's Hospital to find a representative who could bring attention to fundraising for cancer research. They discovered Einar Gustafson, a young boy undergoing treatment for a rare form of lymphoma. To safeguard his privacy, Farber insisted that the boy be identified only as "Jimmy." 'Jimmy' was among the first children to respond positively to the newly discovered antifolate drug. However, 'Jimmy' was diagnosed with Burkitt lymphoma, not acute undifferentiated leukemia, and he was cured. Jimmy evolved as an embodiment of all pediatric cancer cases, and subsequently, the CCRF was renamed 'The Jimmy Fund' [3-7].

During Jimmy's appearance on the radio show, host Ralph Edwards urged listeners to contribute money for the Children's Cancer Research Fund, raising $231,000 throughout an eight-minute broadcast. Philanthropic support poured in, leading to the establishment of the Jimmy Fund Building in the year 1952. The Boston Braves Club, a member of the National Baseball League, selected the Jimmy Fund as the beneficiary of its community-based fundraising initiatives. After the Braves relocated to Milwaukee, the Boston Red Sox, their American League counterparts, designated the Jimmy Fund as their preferred philanthropy [3-7].

The Jimmy Fund and pediatric cancer research received additional support from Harvard University undergraduate students who orchestrated the inaugural figure skating exhibition featuring US national and Olympic champions. In 1969, the CCRF broadened its programs to encompass patients of all ages. In 1998, Einar Gustafson deviated from the traditional selection of mayors, Olympic gold medalists, and other notable figures by throwing the ceremonial first pitch at a Boston Red Sox-New York Yankee game at Fenway Park. He resided and functioned in Maine, working as a truck driver, was blessed with six grandchildren, and passed away in 2001 from a stroke [3-7].

Oncology contributions

Leukemia

In biochemistry, Y. SubbaRow, a distinguished professor at Harvard Medical School, made significant advancements in the synthesis and analysis of folic acid and its derivatives. He collaborated with researchers at Lederle Laboratories, New York, leading to the synthesis of pteroylaspartic acid, a crucial antagonist of various forms of folic acid. SubbaRow's meticulous laboratory experiments and avian trials revealed that pteroylaspartic acid obstructed folate metabolism and hindered normal cellular growth, findings that remain pivotal in the field today [8]. Concurrently, Farber observed the stimulating effects of folate on hematopoietic cells, suggesting that folate antagonists could potentially induce cancer regression. Subsequently, Farber and his medical team in Boston treated 90 patients with different malignancies using pteroylglutamic acid conjugate Aminopterin provided by Y. SubbaRow [9]. Although this 'Phase II' trial did not meet conventional standards of success, certain tumors exhibited accelerated growth followed by regression. It was hypothesized that the regression was associated with a decline in folate levels within rapidly proliferating tumors that had surpassed their vascular supply.

Subsequently, Farber and Louis K. Diamond utilized aminopterin to treat 16 children with acute undifferentiated leukemia between November 1947 and April 1948. Remarkably, temporary remissions, accompanied by clinical, hematological, and bone marrow improvements, were documented in ten patients; most of them were critically ill at the commencement of the therapy. The writers presented detailed clinicopathological information on the most responsive children. Six cases demonstrated no response to the treatment, and four had passed away due to the illness. This event signified the first instance of utilizing a folate antagonist in treating pediatric cases with acute leukemia, representing a significant milestone in medical history [2].

Farber's breakthrough initially received widespread support from medical practitioners but was met with skepticism from many scientists who perceived Farber as being presumptuous. However, Farber's findings represented a significant step forward in cancer research, as previous medications had not demonstrated efficacy against bodily fluid tumors. This work marked a crucial milestone in treating a disease resistant to therapy. Despite criticism from the academic and scientific communities, the paper garnered positive feedback from parents and pediatricians seeking guidance for children or patients with leukemia. This prompted Farber to personally address their inquiries [7].

The discovery of folic acid antagonists as a successful treatment for acute leukemia represented a significant advancement in the field. This discovery paved the way for identifying other potent cytotoxic agents, initially utilized independently and later combined with chemotherapy. By the mid to late 1950s, remission rates for acute lymphoblastic leukemia (ALL), the most prevalent form of cancer in children, had risen. However, during the early 1960s, the median survival for acute leukemia was under one year, with fewer than 1% of children surviving without the illness. Subsequent enhancements in combined chemotherapy techniques led to increased remission rates. Further progress in treatment by including consolidation part and maintenance treatment, the implementation of central nervous system-directed treatment, enhanced supportive care, and the identification of prognostic factors for customizing therapy based on different risks of relapse and mortality have collectively fueled advancements in the treatment of childhood lymphoblastic leukemia. Since the seminal study by Farber et al. nearly 60 years ago, the prevailing disease-free survival has risen from 0% to approximately 80%, marking a distinctive accomplishment [1]. The thriving clinical approach devised for pediatric leukemia has served as a paradigm for cancer chemotherapy.

Wilms Tumor

In the 1950s and 1960s, Farber's groundbreaking research significantly advanced our understanding of cancer. His pioneering discovery in 1955, which showed that combining the antibiotic actinomycin D with postoperative radiation therapy could induce remission in Wilms' tumor, a pediatric kidney cancer, was a major breakthrough [10]. This discovery, made possible by actinomycin D derived from *Streptomyces parvulus *and provided by the Eli Lilly Pharmaceutical Company, proved to be a game-changer in preventing local recurrences and distant metastases in Wilms tumor, contrasting with the prevailing standard therapy involving surgery and radiotherapy to the tumor bed. The incorporation of actinomycin D with lung-targeted radiation therapy effectively eradicated previously treatment-resistant pulmonary metastases, marking a significant leap in cancer treatment [11]. Additionally, actinomycin D's application expanded to treating osteogenic sarcoma [10]. The National Wilms Tumor Study Group achieved an impressive 90% survival rate in children with Wilms' tumors by the end of the century as treatment strategies diversified [12].

In a 1963 interview with Redbook magazine, Farber expressed his optimism about these findings, stating, "Now we are an army on the march [7]." This sentiment was echoed in leading publications such as the Saturday Evening Post, which proclaimed, "Cancer Is Yielding Up Its Secrets [7]." In clinical practice, the once-toxic aminopterin has been overtaken by the comparatively less toxic yet equally efficacious methotrexate. Methotrexate is a pivotal component in the treatment of a broad spectrum of malignancies, encompassing leukemia, lymphoma, gastrointestinal tumors, breast cancer, bone neoplasms, and urogenital, germ cell, and other malignancies [13].

Conception of Comprehensive Care/Total Care

Farber introduced the "total care" concept for cancer patients and their caregivers, which involved integrating clinical care, nutrition, social work, and counseling services in a single location. In the mid-1950s, he advocated for the dedication of an entire floor to inpatient cancer care at CMCH. This approach has become the norm in bigger academic or academic-affiliated pediatric oncology centers across the US. In a 1965 article for CA: A Cancer Journal for Clinicians, he stressed the importance of providing comprehensive support to leukemia patients, even without curative therapy [3-7].

Cancer Research Advocacy

In the early 1950s, Farber advocated for increased funding for cancer research during Congressional hearings. His advocacy played a vital role in creating the Clinical Trials Cooperative Group Program and the Chemotherapy National Service Center within the National Cancer Institute (NCI). Through partnerships with prominent figures like Mary Woodard Lasker, esteemed surgeon Michael E. DeBakey, Senator J. Lister Hill, and Congressman John E. Fogarty, Farber played a pivotal role in boosting federal funding for cancer research. This effort led to a substantial increase in the NCI's budget, which surged from $48 million to $176 million from 1957 to 1967 [3-7].

Additional contributions

Farber made significant contributions to medicine. He made his groundbreaking research in 1938 on sudden infant death syndrome, which garnered widespread attention [14]. His work on the transposition of the great cardiac vessels provided a foundational understanding and played a crucial role in developing pediatric cardiac surgery [15]. Farber also identified the Eastern equine virus as a cause of encephalitis in infants and children and made innovative contributions to the pathology of vitamin deficiencies, offering crucial insights into dihydrotachysterol's effects on experimental rickets in rodents [16,17]. Additionally, in collaboration with Dr. Maddock, Dr. Shwachman, and their team, he significantly contributed to the seminal characterization of cystic fibrosis as a systemic disease. They also defined the enzymatic irregularities linked with celiac disease and pancreatic insufficiency [18,19].

The investigation of lipid disorders, notably Farber disease, has been a significant area of interest since Farber's initial work and subsequent studies by Crocker and Farber in 1958 [20-22]. Farber disease, also known as disseminated lipogranulomatosis or ceramidase deficiency, a rare, autosomal recessive disorder, was first recognized in 1952 [20]. This insufficiency causes the accumulation of ceramide, primarily chondroitin sulfate B, in various organs such as the brain, joints, heart, and kidneys. Unfortunately, Farber disease is relentlessly progressive and generally fatal during early infancy, and no effective therapy has been identified in the fifty years since its original description. Furthermore, in partnership with Farber, hematologists Edmund Klein and Gustave Freeman played a significant role in advancing techniques for isolating platelets from the blood and creating platelet concentrates to support the care of oncology patients [23]. Consequently, Farber's contributions have established him as a foundational figure in pediatric pathology.

Farber, renowned for his work in pediatric pathology, autopsy, and medical history, authored more than 270 influential books and research articles throughout his distinguished career. His 1937 publication, "The Postmortem Examination," significantly influenced autopsy methods and techniques [6].

Memberships and awards

Farber's illustrious career encompassed significant involvement in esteemed organizations, such as the National Advisory Cancer Council, the National Advisory Health Council, and the President's Commission on Heart Disease, Cancer, and Stroke. He served as president of the American Association of Pathologists and Bacteriologists while receiving the esteemed Gold-Headed Cane Award in 1972, a replica of the cane used by physicians serving the British Royal Family between 1689 and 1825. Farber was conferred honorary degrees from several prestigious universities like Suffolk, Boston, Brandeis, Providence, Ghent, Louvain, Karolinska Institute, Albert Einstein College of Medicine, and New York Medical College. His pioneering pediatric chemotherapy work earned him the Albert Lasker Award for Clinical Research in 1966. Additionally, he received accolades such as the Judd Award for research in oncology from the Memorial Sloan Kettering Cancer Center and the Variety Club's International Humanitarian Award. Farber's influential consulting roles included positions with respected organizations like the Armed Forces Institute of Pathology and the US Public Health Service. Furthermore, his status as a founding trustee of the United States Cerebral Palsy Research and Educational Foundation and his election as President of the American Cancer Society in 1968 underscore the breadth of his contributions. Farber Hall, erected in 1953 on the South Campus of the State University of New York Buffalo, stands as a lasting tribute to his memory [3-7].

In 1974, the CCRF was renamed the Sidney Farber Cancer Center, and in 1976, it was designated as the Sidney Farber Cancer Institute. The Charles A. Dana Building was constructed in 1978 and funded generously by industrialist Charles A. Dana and his foundation, which is a testament to their support. The institute was formally rebranded as the Dana-Farber Cancer Institute (DFCI) in 1983. Today, the DFCI stands as a globally renowned clinical and laboratory cancer research center, a testament to Sidney Farber's legacy and a source of pride for the medical community [6,7].

## Conclusions

Farber pioneered cancer treatment with his immense contributions to leukemia and Wilms tumor treatment. He was always innovative and pioneered the introduction of crucial visions in pediatric oncology, including the hospital day-care settings, comprehensive multidepartmental involvement in cancer care involving social workers, nutritionists, psychological aid, and multidepartmental involvement in tumor boards to discuss issues and devise diagnostic and therapeutic strategies. Despite being a pathologist and not directly involved in clinical care, Farber's innovative approaches have substantially impacted cancer care.

Farber's realization of the marketing significance in furthering scientific understanding of diseases was prompted by the successful evolution of the Jimmy Fund. Throughout his career, Farber expanded his expertise from pathology and oncology to encompass clinical practice and advocacy for cancer research. This evolution mirrored the shifting societal attitudes toward cancer and played a crucial role in driving increased funding and attention for cancer research throughout the rest of the century. Nonetheless, the research discoveries attributed to Farber have established him as the eminent figure recognized as the 'Founder of Pediatric Pathology' and the 'Father of Modern Chemotherapy.'
